# Addressing male sexual and reproductive health in the wake of COVID-19 outbreak

**DOI:** 10.1007/s40618-020-01350-1

**Published:** 2020-07-13

**Authors:** A. Sansone, D. Mollaioli, G. Ciocca, E. Limoncin, E. Colonnello, W. Vena, E. A. Jannini

**Affiliations:** 1 Chair of Endocrinology and Medical Sexology (ENDOSEX), Department of Systems Medicine, University of Rome Tor Vergata, via Montpellier 1, 00133 Rome, Italy; 2 Department of Dynamic and Clinical Psychology, “Sapienza” University of Rome, Rome, Italy; 3 Endocrinology, Diabetology and Andrology Unit, Humanitas Clinical and Research Center, IRCCS, Rozzano, MI Italy; 4 Department of Clinical Sciences and Community Health, University of Milan, Milano, Italy

**Keywords:** COVID-19, SARS-CoV-2, Erectile dysfunction, Sexual dysfunction, Male hypogonadism, Cardiovascular health

## Abstract

**Purpose:**

The COVID-19 pandemic, caused by the SARS-CoV-2, represents an unprecedented challenge for healthcare. COVID-19 features a state of hyperinflammation resulting in a “cytokine storm”, which leads to severe complications, such as the development of micro-thrombosis and disseminated intravascular coagulation (DIC). Despite isolation measures, the number of affected patients is growing daily: as of June 12th, over 7.5 million cases have been confirmed worldwide, with more than 420,000 global deaths. Over 3.5 million patients have recovered from COVID-19; although this number is increasing by the day, great attention should be directed towards the possible long-term outcomes of the disease. Despite being a trivial matter for patients in intensive care units (ICUs), erectile dysfunction (ED) is a likely consequence of COVID-19 for survivors, and considering the high transmissibility of the infection and the higher contagion rates among elderly men, a worrying phenomenon for a large part of affected patients.

**Methods:**

A literature research on the possible mechanisms involved in the development of ED in COVID-19 survivors was performed.

**Results:**

Endothelial dysfunction, subclinical hypogonadism, psychological distress and impaired pulmonary hemodynamics all contribute to the potential onset of ED. Additionally, COVID-19 might exacerbate cardiovascular conditions; therefore, further increasing the risk of ED. Testicular function in COVID-19 patients requires careful investigation for the unclear association with testosterone deficiency and the possible consequences for reproductive health. Treatment with phosphodiesterase-5 (PDE5) inhibitors might be beneficial for both COVID-19 and ED.

**Conclusion:**

COVID-19 survivors might develop sexual and reproductive health issues. Andrological assessment and tailored treatments should be considered in the follow-up.

## Introduction

The global outbreak of coronavirus disease (COVID-19) caused by the severe acute respiratory syndrome coronavirus 2 (SARS-CoV-2) represents an unprecedented challenge for healthcare. Despite social distancing and isolation measures, the number of affected patients is growing daily. Hyperinflammation and immunosuppression are prominently featured in COVID-19 [1, 2], resulting in a cytokine storm [3] ultimately leading to development of micro-thrombosis and disseminated intravascular coagulation (DIC). This cytokine storm is strongly associated with the development of interstitial pneumonia (IP) [4]; however, although lungs are the primarily targeted organs, the cardiovascular system is globally affected. Evidence in this regard supports the notion that the exaggerated production of early response proinflammatory cytokines, such as tumor necrosis factor (TNF), interleukin-1β, -6, and -10 (IL-1β, IL-6, and IL-10, respectively), increases the risk of vascular hyperpermeability, possibly progressing to multiple organ failure and, ultimately, death [3]. The presence of vascular dysfunction at multiple levels, including pulmonary embolisms, alveolar hemorrhage, microangiopathy and vasculitis has been ascertained in post-mortem examination [5, 6]. Additionally, both venous and arterial thromboembolic complications, including endothelial inflammation, have been reported [7, 8]. Indeed, a growing body of evidence seems to support the theory that the endothelium is targeted by the SARS-CoV-2 [9]; most importantly, the endothelium expresses the protein angiotensin-converting enzyme 2 (ACE2) [10, 11], through which the virus can access host cells [12]. Endothelial dysfunction is, therefore, a pivotal determinant of COVID-19 symptoms [13, 14].

As of June 12th, 2020, more than 7.5 million COVID-19 cases have been confirmed worldwide, with more than 420,000 lives lost due to the disease [15]. More than 3.5 million subjects have recovered from COVID-19; however, the long-term consequences of the disease are still largely unknown. Data from 2002–2004 epidemics of SARS suggest that cardiovascular sequelae, such as microangiopathy, cardiomyopathy and impaired endothelial function, are to be expected also in COVID-19 patients [5, 16]. However, while similarities with SARS have been identified, COVID-19 is largely more prevalent due to its high transmissibility, and its consequences, even for recovered patients, are likewise more worrying. Additionally, new evidence is suggesting that autoimmune conditions, such as type 1 diabetes mellitus, might be triggered by the onset of COVID-19 [17], therefore, worsening the risk profile for survivors.

These findings can be extremely relevant for male sexual health: indeed, based on these premises, there is quite enough evidence to hypothesize that consequences of COVID-19 can extend to sexual and reproductive health. We investigated the current literature to understand the long-term clinical complications for COVID-19 survivors, aiming to provide adequate information for clinicians to plan adequate and timely intervention measures.

### Testosterone and COVID-19: friend or foe?

It is well established that ACE2 is the entry point for the SARS-CoV-2 in host cells [12]. In males, adult Leydig cells express this enzyme, therefore, suggesting that testicular damage can occur following infection [18]. Testicular damage in COVID-19 might, therefore, induce a state of hypogonadism as proven by decreased testosterone-to-LH ratio in patients with COVID-19, suggestive of impaired steroidogenesis resulting from subclinical testicular dysfunction [19, 20]. Post-mortem examinations of testicular tissue from 12 COVID-19 patients showed significantly reduced Leydig cells, as well as edema and inflammation in the interstitium [21]. A recent report on 31 male COVID-19 patients in Italy identified that some patients developed hypergonadotropic hypogonadism following the onset of the disease [22]. In the same study, lower levels of serum testosterone (total and free) acted as predictors of poor prognosis in SARS-CoV-2 men [22]. Whether this state of hypogonadism is permanent or temporary is a question so far left unanswered. Testosterone acts as a modulator for endothelial function [23] and suppresses inflammation by increasing levels of anti-inflammatory cytokines (such as IL-10) and reducing levels of pro-inflammatory cytokines such as TNF-α, IL-6 and IL-1β [24]. It can, therefore, be hypothesized that suppression of testosterone levels might be one of the reasons for the large difference in terms of mortality and hospitalization rate between males and females and might also explain why SARS-CoV-2 most commonly infects old men.

On the other hand, androgens seem to play a pivotal role in COVID-19 by promoting the transcription of the transmembrane protease, serine 2 (TMPRSS2) gene. The encoded protein primes the spike protein of SARS-CoV-2, therefore, impairing antibody response and facilitating the fusion between the virus and the host cells [25]. This hypothesis could explain the higher prevalence of COVID-19 in men, although it would fail to explain the rationale for the higher mortality rates, as well as the worse clinical outcomes, for elderly patients.

Additional studies would, therefore, be needed to understand whether testosterone treatment might be beneficial or deleterious for the clinical course of the disease. However, independently of whether testosterone is a friend or foe for COVID-19, it should be acknowledged that the testis is a target for SARS-CoV-2 and the possibility for long-lasting consequences on the endocrine function exists, even for recovered patients.

### COVID-19 and the endothelium

Solid evidence accumulated in the last decades support the notion that erectile function is an excellent surrogate marker of systemic health in general, and vascular performance in particular [26], sharing plenty of risk factors with cardiovascular disease. This is described by the equation ED = ED (endothelial dysfunction equals erectile dysfunction, and vice versa) [27]. Vascular integrity is necessary for erectile function [28], and vascular damage associated with COVID-19 is likely to affect the fragile vascular bed of the penis, resulting in impaired erectile function [5, 7]. COVID-19 features a state of hyperinflammation promoted by TNF-α, IL-6 and IL-1β [29]; the same inflammatory cytokines have been associated with clinical progression of sexual dysfunction [30]. It is worth noticing that the pro-inflammatory cytokines are also closely tied to testosterone levels: as previously stated, hypogonadal patients have higher concentrations of TNF-α, IL-6 and IL-1β as a result of impaired suppression. This ultimately worsens the endothelial dysfunction, further impairing erectile function. However, whether testosterone replacement therapy (TRT) would improve endothelial function is still debated, while largely beneficial in the treatment of hypogonadal men, TRT has known harmful effects if inappropriately prescribed [31], and a meta-analysis study did not find any conclusive evidence of a potentially therapeutic effect of testosterone administration, neither acute nor chronic, on endothelial function [32]. While erection is—of course—a trivial matter for patients in Intensive Care Units (ICUs), there is reason to suspect that impaired vascular function might persist in COVID-19 survivors and even become a public health issue in the next few months. Moreover, given that erectile function is a predictor of heart disease [33, 34], investigating whether COVID-19 patients develop ED might also be a good surrogate marker of general cardiovascular function, improving patient care and quality of life.

### A COVID eclipse of the heart: potential for cardiovascular burden

Besides the effects on endothelium, SARS-CoV-2 infection can also dramatically affect the heart and exacerbate underlying cardiovascular conditions. Reports of myocarditis in COVID-19 patients have piled up in the last months [35–37]; similarly, arrhythmias and acute cardiovascular events have been described in other coronavirus and influenza epidemics [38–40] and are likely to be expected for SARS-CoV-2 as well [41]. COVID-19 survivors are, therefore, more likely to develop severe cardiovascular consequences. However, treatment is not exempt from possible side effects, among which sexual dysfunctions are remarkably common. Drugs such as β-blockers and antihypertensive agents, routinely used in COVID-19 patients, have the potential to impair sexual function [41]; therefore, both the cardiovascular consequences and their treatment might ease progression from subclinical to a clinically overt ED [42, 43].

It is also worth mentioning that several cardiovascular risk factors involved in sexual dysfunctions, such as smoking [44], diabetes [45] and hyperhomocysteinemia [46–49], are also possible predictors of worse outcomes in COVID-19 patients.

Additionally, as stated in the III Princeton Consensus Panel [50], sexual activity should be delayed until the cardiac condition has been stabilized in high-risk patients. Such patients include those with uncontrolled hypertension, recent myocardial infarction or high-risk arrhythmia, which are all conditions closely associated with COVID-19 [51].

### Reproductive health and COVID-19

Another reason for worry lies in the reported testicular damage from COVID-19 infection. In fact, ACE2 is highly expressed in the testis, suggesting the possibility of testicular infection since the early stage of the disease [52]. Being expressed in both Sertoli and Leydig cells [18, 53], ACE2 plays key roles in spermatogenesis and in the regulation of steroidogenesis. Due to the involvement of Sertoli cells, reproductive function might similarly be affected. Additionally, ACE2 is also expressed by spermatogonia, therefore, increasing the risk of SARS-CoV-2 presence in seminal fluid [54, 55].

Studies investigating the presence of SARS-CoV-2 in seminal fluid have, for the largest part, found no evidence of the virus [56–59]. However, as other studies have shown different results [60], the topic of reproductive health is still largely debated. In post-mortem examinations, seminiferous tubular injury was reported despite no evidence of the virus in the testis [21]. Identification of SARS-CoV-2 in semen is of the utmost importance, as sperm cryopreservation is an undelayable necessity for many men, such as those who are about to start gonadotoxic treatments [61]. In Italy, cryopreservation procedures for oncological patients have continued during the COVID-19 pandemic, using utmost care to limit the risk of transmission; for non-oncological patients, the prospects of biological parenthood could be compromised as a consequence of delaying diagnostic semen analysis and sperm banking [62]. At the beginning of the pandemic, discontinuation of reproductive care except was recommended by international societies for reproductive medicine, with only the most urgent cases allowed; as containment and safety strategies have mitigated the spread of the disease, several centers for assisted reproductive technology have resumed their activity, although with very precise rules for operators [63, 64].

Further studies should, therefore, be designed with the aim to clarify this point, above all among “COVID-19 asymptomatic” men requiring assisted reproductive technology (ART).

### The psychological burden of COVID-19

Increased rates of post-traumatic stress disorder (PTSD), depression and anxiety are expected in the general population, and even more in COVID-19 survivors, following the pandemic [65–68]. A parallel can be drawn between the psychological consequences of COVID-19 and those coming from similar disasters, such as the 9/11 attacks [69] or earthquakes [70], and similar short- and long-term treatment strategies are, therefore, needed to provide adequate care. Confinement and the illness in itself are both causes of stress; while only a minority of individuals might be more vulnerable to psychological trauma, there is no doubt that most people would experience some degree of emotional distress following isolation, social distancing, loss of relatives and friends, difficulties in securing medications, as well as the obvious economic consequences of lockdown. Sexual activity is closely associated with mental and psychological health; it is, therefore, unsurprising that sexual desire and frequency have declined in both genders during this pandemic [71, 72]. There is, therefore, reason to suspect that psychological suffering might exacerbate pre-existing subclinical sexual dysfunctions [73]. Additionally, the potential for SARS-CoV-2 transmission by kissing might lead to increased distress in the couple [74], with the resulting negative effects on sexual health and on couple dynamics. Additionally, the hypogonadal state reported in COVID-19 could lead to a significant worsening in sexual desire and mood [75, 76].

### Pulmonary fibrosis and the effects of hypoxia

It has been suggested, with on the basis of interesting evidence, that there could be substantial fibrotic consequences following SARS-CoV-2 infection [77, 78]. Indeed, pulmonary fibrosis is a well-acknowledged consequence of acute respiratory distress syndrome (ARDS), with further evidence coming from survivors of the 2003 SARS outbreak (caused by the SARS-CoV) [79, 80]. Pulmonary fibrosis impairs the physiologic lung mechanisms, reducing the pulmonary gas exchange and, therefore, impairing oxygen saturation [81, 82]; functional disability has been proven in ARDS patients several years after the acute phase of the disease [83]. There is currently no evidence concerning the possible long-term impairment of lung function following SARS-CoV-2 infection; however, considering the scale of the current pandemic and the similarities between SARS-CoV and SARS-CoV-2 [84], there is sufficient reason to suspect a high rate of fibrotic lung function abnormalities in COVID-19 survivors. In such patients, the impaired oxygen saturation could impair erectile function; some evidence in support comes from animal models [85, 86] as well as from clinical reports [87, 88]. From a pathophysiological standpoint, this is hardly surprising, as oxygen is one of the substrates required for the synthesis of nitric oxide (NO) by the enzyme NO synthase, whose activity is severely blunted in hypoxia [87].

### Phosphodiesterase-5 inhibitors in COVID-19

Phosphodiesterase-5 (PDE-5) belongs to the PDE superfamily of enzymes, the last step of the NO/cGMP/PDE pathway and is one of the key elements in drug treatment of ED. NO activates guanylate cyclase in responsive cells, such as endothelial cells, resulting in increased concentrations of the second messenger cGMP (cyclic guanosine monophosphate), which in turn induces relaxation of smooth muscle. PDE acts downstream and reduces effects of cGMP by catalyzing its degradation: PDE inhibitors prevent degradation of cGMP, resulting in prolonged or enhanced action [89].

PDE-5 is highly expressed in vascular smooth muscle cells [90], and, at high concentrations, in those of the penile corpora cavernosa [91]; therefore, thanks to their action and due to their high affinity for the specific type 5 isoform [92], PDE-5 inhibitors have been approved for their use in treatment of ED since 1998. However, a growing body of evidence has also proven their usefulness as therapeutic agents in different conditions due to their anti-inflammatory and antioxidant actions, as reported in diabetes [93], hypertension and chronic kidney disease [94]. Sildenafil, the first PDE-5 inhibitor approved for the treatment of ED following its serendipitous discovery [95], has also been investigated as a treatment for COVID-19 patients; indeed, Sildenafil improves pulmonary hemodynamics, as shown in idiopathic pulmonary fibrosis [96], by reducing vascular resistance and remodeling in the pulmonary circulation [97]. Additionally, by inhibiting neointimal formation and platelet aggregation, sildenafil also might prove beneficial in regard to the risk of vascular injury and thrombotic complications in COVID-19 patients [98]. Evidence from new trials will prove fundamental to assess the clinical benefits of PDE-5 inhibition on the overall burden of COVID-19 [99].

## Conclusions

**Fig. 1:**
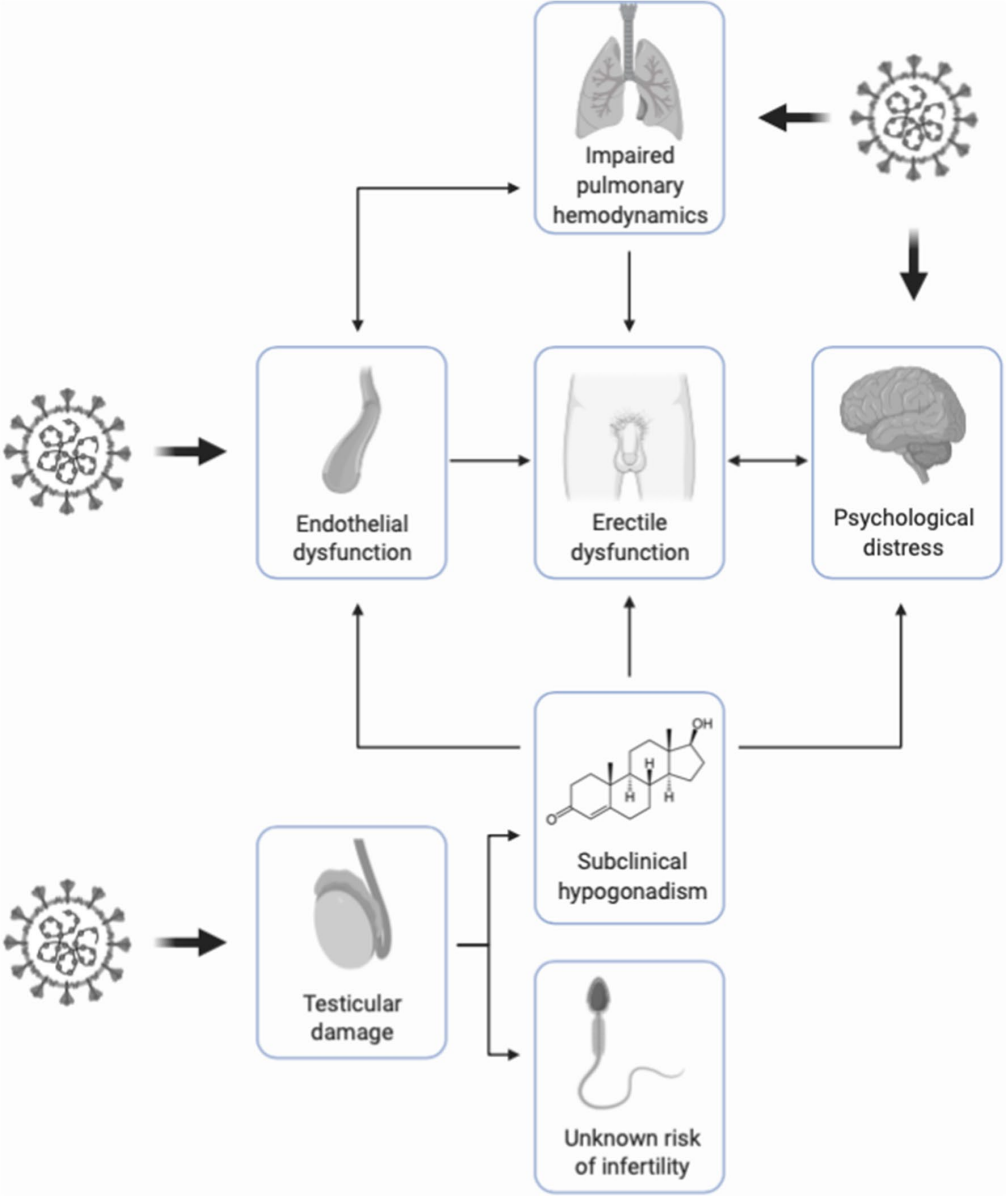
Graphical overview of the involvement of SARS-CoV-2 in the pathogenesis of erectile dysfunction

In conclusion, there is quite enough reason to suspect that male sexual and reproductive health could be affected in the survivors, by the sequelae of the COVID-19, both in the short and long terms (Fig. 1). Erectile function, as a surrogate marker of cardiovascular/pulmonary health, could also become extremely valuable as a quick and inexpensive first-line assessment of the pulmonary and cardiovascular complications for COVID-19 survivors. In this regard, evidence coming from diagnostic procedures, such as penile color-doppler ultrasound [43] and hypothalamic-pituitary–testicular axis evaluation [100], will be necessary to assess the extent to which COVID-19 has been able to impair erectile, and finally vascular, function, the former being an efficient predictor of complete *restitutio ad integrum*. Additionally, tailored psychological interventions would be necessary to adequately support patients who develop sexual dysfunction consequently to the containment measures.
